# Optimizing survival outcomes with post‐remission therapy in acute myeloid leukemia

**DOI:** 10.1002/ajh.25484

**Published:** 2019-05-01

**Authors:** Bruno C. Medeiros, Steven M. Chan, Naval G. Daver, Brian A. Jonas, Daniel A. Pollyea

**Affiliations:** ^1^ Department of Hematology, Stanford University School of Medicine Stanford California; ^2^ Princess Margaret Cancer Centre Toronto Ontario Canada; ^3^ Department of Leukemia MD Anderson Cancer Center Houston Texas; ^4^ University of California Davis Comprehensive Cancer Center Sacramento California; ^5^ University of Colorado School of Medicine Aurora Colorado

## Abstract

Optimization of post‐remission therapies to maintain complete remission and prevent relapse is a major challenge in treating patients with acute myeloid leukemia (AML). Monitoring patients for measurable residual disease (MRD) is helpful to identify those at risk for relapse. Hypomethylating agents are being investigated as post‐remission therapy. Identification of recurrent genetic alterations that drive disease progression has enabled the design of new, personalized approaches to therapy for patients with AML. Emerging data suggest that targeted post‐remission therapy, alone or in combination with chemotherapy, may improve outcomes. Results of ongoing clinical trials will further define potential clinical benefits.

## 1 INTRODUCTION

In adults with newly diagnosed acute myeloid leukemia (AML), complete remission (CR) generally can be achieved in up to 70% of younger patients (aged <60 years), and in up to 50% of older patients (aged ≥60 years) with conventional high‐intensity induction regimens.^1^ Despite these relatively high rates of initial response, relapse rates range from approximately 30% to 35% in younger patients with favorable risk factors, to 70% to 80% in older patients with adverse risk factors.^2^ The 5‐year overall survival (OS) rate ranges from 40% to 50% in patients aged <60 years and from 20% to 30% in patients aged >60 to 70 years who receive high‐intensity chemotherapy regimens.^3^ Furthermore, in a study of 2551 patients with AML who did not undergo stem cell transplant, the 10‐year progression‐free survival (PFS) rate was 2.4% among patients aged ≥60 years.^4^

Post‐remission therapy (consolidation and maintenance therapy) for AML aims to maintain and/or prolong remission by eliminating residual leukemic cells and preventing relapse.^1^ Categorizing patients by risk status based on validated cytogenetic and molecular abnormalities (such as mutation status of the nucleophosmin gene [*NPM1*], the CCAAT/enhancer binding protein α gene [*CEBPA*], and the FMS‐like tyrosine kinase receptor 3 [*FLT3*] gene internal tandem duplication [ITD]) guides treatment decisions with regard to post‐remission therapy.^1^, ^5^ The National Comprehensive Cancer Network (NCCN) Clinical Practice Guidelines for AML^1^ state that karyotype is the most important prognostic factor for rates of remission, risk of relapse, and OS. The incorporation of molecular abnormalities further refines risk stratification, and identifies potential targeted therapeutic approaches at all stages of treatment. A personalized post‐remission treatment strategy that is tailored based on overall risk and specific genetic alterations will be key to improving clinical outcomes.^1^, ^5^

The current approach to post‐remission therapy is for patients with relatively low risk of relapse to undergo successive cycles of chemotherapy alone, and for higher risk patients to undergo hematopoietic cell transplantation (HCT). Allogeneic HCT is typically recommended for patients with high‐risk features (including intermediate‐ and adverse‐risk genomic abnormalities), after salvage therapy, and for patients with secondary AML. Although the use of reduced‐intensity conditioning regimens has enabled more patients with high‐risk AML to undergo allogeneic HCT,^6^ it may also contribute to higher rates of post‐transplant relapse.^7^ Unfortunately, relapses occur in both high‐risk and low‐risk patients, and outcomes are generally very poor for patients who experience relapse.^8^ Therefore, better post‐remission therapies are needed to prevent relapses and improve long‐term survival. As such, optimization of therapy post remission represents a major challenge in the treatment of patients with AML, and the role of maintenance therapy has remained somewhat controversial and inadequately tested.

The objectives of this article are to discuss factors that affect relapse in patients with AML, review non‐allogeneic HCT post‐remission strategies, and describe potential new approaches under investigation.

## 2 POST‐REMISSION TREATMENT APPROACHES

### 2.1 Consolidation therapy

The NCCN Guidelines for AML provide recommendations for post‐remission therapy based on age (<60 or ≥60 years; Table 1) and risk of relapse by cytogenetics and molecular abnormalities.^1^ Post‐remission therapy recommendations from the European LeukemiaNet (ELN) differ somewhat from those of the NCCN. ELN‐recommended consolidation therapy is determined by both age (younger patients [18‐60/65 years] and older patients [>60/65 years]) and genetic risk (cytogenetic and molecular; Table 1).^5^ Over the past 12 months, two targeted therapies were approved for use in induction and consolidation therapy for patients with AML: the FLT3 tyrosine kinase inhibitor, midostaurin (see section 2.2), and the antibody‐drug conjugate targeting CD33, gemtuzumab ozogamicin. In addition, a liposomal formulation of daunorubicin and cytarabine was approved for induction and consolidation therapy for untreated AML patients with high‐risk features.

**Table 1:** Summary of National Comprehensive Cancer Network^1^ and European LeukemiaNet Guidelines for post‐remission therapy in patients with AML^5^

National Comprehensive Cancer Network	
Patients aged <60 y with favorable risk	• HiDAC 3 g/m^2^ over 3 h every 12 h on days 1, 3, 5 or 1, 2, 3 × 3‐4 cycles, or • Cytarabine 1000 mg/m^2^ every 12 h on days 1‐4 + daunorubicin 60 mg/m^2^ on day 1 (first cycle) or days 1‐2 (second cycle) + gemtuzumab ozogamicin 3 mg/m^2^ on day 1 × 2 cycles (CD33‐positive)
Patients aged <60 y with intermediate risk	Matched sibling or alternative donor HCT, or HiDAC 1.5‐3 g/m^2^ over 3 h every 12 h on days 1, 3, 5 or 1, 2, 3 × 3‐4 cycles, or HiDAC 1.5‐3 g/m^2^ over 3 h every 12 h on days 1, 3, 5 or 1, 2, 3 with oral midostaurin 50 mg every 12 h on days 8‐21 (*FLT3*‐mutated AML), or Cytarabine 1000 mg/m^2^ every 12 h on days 1‐4 + daunorubicin 60 mg/m^2^ on day 1 (first cycle) or days 1‐2 (second cycle) + gemtuzumab ozogamicin 3 mg/m^2^ on day 1 × 2 cycles (CD33‐positive)
Patients aged <60 y with treatment‐related disease other than CBF and/or with poor risk	Matched sibling or alternative donor HCT, or HiDAC 1.5‐3 g/m^2^ every 12 h on days 1, 3, 5 or 1, 2, 3 × 3‐4 cycles, or HiDAC 1.5‐3 g/m^2^ every 12 h on days 1, 3, 5 or 1, 2, 3 with oral midostaurin 50 mg every 12 h on days 8‐21 (*FLT3*‐mutated AML), or Dual‐drug liposomal encapsulation cytarabine 65 mg/m^2^ and daunorubicin 29 mg/m^2^ on days 1 and 3 (cytotoxic therapy‐related AML or patients with antecedent MDS/CMML or cytogenetic changes consistent with MDS)
Patients aged ≥60 y with CR after intensive induction therapy	Reduced‐intensity HCT, or Standard‐dose cytarabine with or without an anthracycline (idarubicin or daunorubicin) or intermediate‐dose cytarabine for 4‐6 doses for 1 or 2 cycles (if good performance status, normal renal function, better‐risk or normal karyotype and favorable molecular markers), or Dual‐drug liposomal encapsulation cytarabine 65 mg/m^2^ and daunorubicin 29 mg/m^2^ on days 1 and 3 (cytotoxic therapy‐related AML or patients with antecedent MDS/CMML or cytogenetic changes consistent with MDS), or Cytarabine 1000 mg/m^2^ every 12 h on days 1‐4 + daunorubicin 60 mg/m^2^ on day 1 (first cycle) or days 1‐2 (second cycle) + gemtuzumab ozogamicin 3 mg/m^2^ on day 1 × 2 cycles (CD33‐positive), or Maintenance therapy with hypomethylating agents (5‐azacitidine, decitabine) every 4‐6 weeks until progression (if patient received hypomethylating agents during induction)
Patients aged ≥60 y with CR after lower intensity therapy	Reduced‐intensity HCT Hypomethylating agents (5‐azacitidine or decitabine) every 4‐6 weeks until progression Gemtuzumab ozogamicin 2 mg/m^2^ on day 1 every 4 weeks up to 8 continuation courses (CD33‐positive) Continue enasidenib (*IDH2*‐mutated AML) or ivosidenib (*IDH1*‐mutated AML) until progression

European LeukemiaNet	
Younger patients (18–60/65 y)	
Favorable risk genetics	2 to 4 cycles of IDAC (1000‐1500 mg/m^2^ every 12 h, days 1‐3 or days 1‐5 or 6) additional therapy Midostaurin for patients with *FLT3* mutations (administered after the chemotherapy)
Intermediate risk genetics	Allogeneic HCT from matched‐related or unrelated donor, or 2 to 4 cycles of IDAC, or High‐dose therapy and autologous HCT Midostaurin for patients with *FLT3* mutations (administered after the chemotherapy)
Adverse risk genetics	Allogeneic HCT from matched‐related or unrelated donor Midostaurin for patients with *FLT3* mutations (administered after the chemotherapy)
Older patients (>60/65 y)	
Favorable risk genetics	2 to 3 cycles of IDAC (500‐1000 mg/m^2^ every 12 h, days 1‐3 or days 1‐5 or 6)
Intermediate/adverse risk genetics	No established value; consider allogeneic HCT with a low HCT‐Comorbidity Index, or investigational therapy

Abbreviations: AML, acute myeloid leukemia; CBF, core‐binding factor; CMML, chronic myelomonocytic leukemia; CR, complete remission; HiDAC, high‐dose cytarabine; HCT, hematopoietic cell transplant; IDAC, intermediate dose cytarabine; MDS, myelodysplastic syndrome.

Gemtuzumab ozogamicin is approved in combination with daunorubicin and cytarabine for the treatment of adults with newly diagnosed AML whose tumors express the CD33 antigen.^9^ In the randomized, open‐label, phase 3 ALFA‐0701 trial (the basis for approval), previously untreated patients aged 50 to 70 years who achieved CR to standard induction therapy, with or without gemtuzumab ozogamicin, received two consolidation courses of daunorubicin, with or without gemtuzumab ozogamicin. Patients with CR who received gemtuzumab ozogamicin had a significantly higher rate of relapse‐free survival (RFS) at two years than controls (50.3% vs 22.7%, respectively; *P* = .0003).^10^ In contrast, in the ECOG E1900 randomized phase 3 trial, 307 patients aged 17 to 60 years with AML who achieved CR after induction therapy (daunorubicin + cytarabine) were randomized to intensive consolidation therapy with or without gemtuzumab ozogamicin (single 6 mg/m^2^ dose) before autologous HCT. The single dose of gemtuzumab ozogamicin did not demonstrate a disease‐free survival (DFS) or OS benefit over control.^11^

CPX‐351, a liposomal formulation of daunorubicin and cytarabine, is approved for adults with newly diagnosed therapy‐related AML or AML with myelodysplasia‐related changes.^12^ In a randomized, open‐label, phase 3 trial, patients aged 60 to 75 years with newly diagnosed secondary AML were randomized to receive up to two cycles of induction with CPX‐351. This was followed by up to two cycles of CPX‐351 consolidation (n = 153), or up to two cycles of conventional cytarabine/daunorubicin 7 + 3 induction, and up to two cycles of 7 + 3 consolidation (n = 156). At a median follow‐up of 20.7 months, median OS was significantly longer in the CPX‐351 group vs the 7 + 3 group (9.56 vs 5.95 months, respectively; *P* = .003).^13^

### 2.2 Maintenance therapy

**Figure 1:**
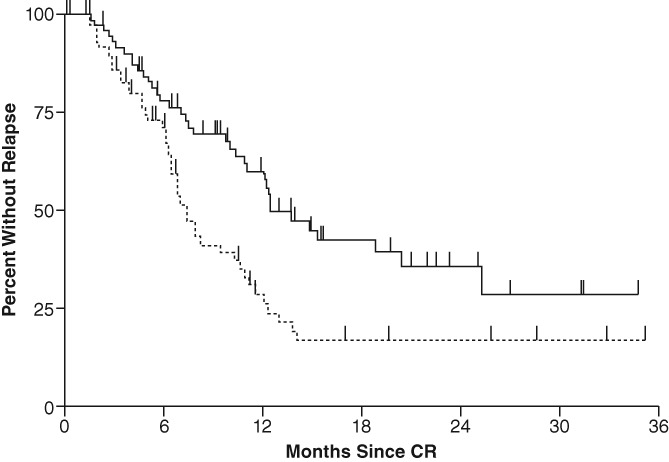
Life‐table analysis of duration of remission following induction, then consolidation, with (dark line; n = 71) and without (hatched line; n = 74) long‐term monthly maintenance in patients with AML. Reprinted with permission from Buchner T, Urbanitz D, Hiddemann W, et al. Intensified induction and consolidation with or without maintenance chemotherapy for acute myeloid leukemia (AML): two multicenter studies of the German AML Cooperative Group. *J Clin Oncol*. 1985;3(12):1583‐1589^15^

The role and benefit of maintenance therapy in adult AML were evaluated more than 30 years ago. This was a trial in which patients were randomized to receive either no further therapy, or long‐term maintenance chemotherapy following a morphologic CR. Patients who received no further therapy experienced a significantly shorter duration of remission compared with patients who received maintenance chemotherapy. They ultimately relapsed in a median of 4.1 months, whereas patients who received maintenance chemotherapy relapsed in a median of 8.1 months (*P* ≤ .002, log rank).^14^ The role of maintenance therapy was tested in a parallel trial, in which previously untreated patients with AML in CR after induction therapy were randomized to receive consolidation therapy, with or without monthly chemotherapy‐based maintenance therapy. This was given until relapse or a maximum of 3 years. Patients who received both consolidation and maintenance therapy had significantly better outcomes (median duration of remission of 13 months and 30% continuous remissions at 2.5 years) compared with patients who received consolidation therapy and no maintenance therapy (median duration of remission of 8 months and 17% continuous remissions at 2.5 years); *P* = .003; Figure 1).^15^

Since these initial observations, a number of regimens have been evaluated as maintenance therapies in patients with AML. In the randomized, double‐blind, placebo‐controlled phase 3 RATIFY trial, oral midostaurin was added to consolidation therapy with cytarabine and was given as single‐agent maintenance therapy. After a median follow‐up of 59 months, results showed that midostaurin significantly prolonged OS vs placebo (median, 74.7 months vs 25.6 months, respectively; *P* = .009), and decreased the risk of death by 22% (*P* = .009). The 4‐year OS rate was 51.4% in the midostaurin group vs 44.3% in the placebo group. Based on these findings, midostaurin is approved by the US Food and Drug Administration (FDA) for the treatment of adults with *FLT3* mutation‐positive AML in combination with standard cytarabine consolidation. Although a subset of patients in the trial underwent HCT, and trial therapy was discontinued at the time of transplantation, the risk of death was 24.3% lower with midostaurin vs placebo after censoring data from patients who underwent transplantation. The 4‐year OS rate was 63.7% in the midostaurin group vs 55.7% in the placebo group in this subset, which was not statistically significant.^16^ The FDA did not approve midostaurin as maintenance therapy because the design of the trial did not allow for determination of the independent effect of maintenance therapy. However, in Europe, midostaurin is approved for the treatment of newly diagnosed patients with *FLT3*‐mutated AML in combination with standard daunorubicin/cytarabine induction followed by high‐dose cytarabine (HiDAC) consolidation, and as single‐agent maintenance therapy for patients in complete response.^17^, ^18^ In an unplanned subset analysis of 174 patients in CR at the start of maintenance therapy, no difference was observed in DFS and OS between the midostaurin and placebo arms during 12 cycles of maintenance therapy.^19^

A prospective phase 3 study compared low‐dose cytarabine (LDAC) maintenance therapy vs observation in 86 patients with AML, aged 18 to 70 years, who achieved CR to induction therapy with HiDAC and amsacrine (when given after relapse or refractoriness to prior induction therapy). LDAC maintenance therapy (10 mg/m^2^ every 12 hours x 21 days every 8 weeks) was initiated within 1 to 3 weeks of CR. Although the regimen was tolerable, and leukemia‐free survival was significantly prolonged with LDAC vs observation (7.7 months vs 3.1 months, respectively; *P* = .027), OS was not significantly prolonged (10.8 months vs 7.0 months, respectively; *P* = NS).^20^

The role of hypomethylating agents as maintenance therapy in AML remains under investigation. In a recent randomized phase 3 study (HOVON 97), post‐consolidation maintenance therapy with azacitidine (50 mg/m^2^ × 5 days every 4 weeks for up to 12 cycles) was compared with observation (control) in patients aged ≥60 years. The patients had AML, or myelodysplastic syndrome (MDS) with excess of blasts and were in CR after ≥2 cycles of intensive chemotherapy. In 115 patients analyzed, maintenance therapy with azacitidine improved 12‐month DFS over control (64% vs 42%, respectively; *P* = .04). After censoring data from patients who received allogeneic HCT, the investigators determined that the 12‐month OS rate was 82% in the azacitidine group and 63% in the control group (*P* = .209).^21^ A phase 2 study with azacitidine (60 mg/m^2^ × 5 days every 28 days as maintenance therapy initiated within 28 days of CR to standard induction chemotherapy) was conducted. The study had 23 patients aged ≥60 years with high‐risk MDS, or AML following MDS, and showed no DFS or OS benefit.^22^ Also, assessment of survival with decitabine (20 mg/m^2^ × 5 days every 6 weeks for 8 cycles) in patients aged <60 years with AML in CR after consolidation therapy showed no benefit in terms of DFS and OS when compared with historical controls.^23^

Thus, the use of maintenance therapy remains controversial. A 2016 systematic literature review of 50 studies assessed the available evidence for the use of maintenance therapy after consolidation therapy or HCT.^24^ At the time of publication, the authors did not recommend maintenance therapy after adequate induction and consolidation therapy or after allogeneic HCT, with the exceptions of clinical trial investigation and the use of midostaurin for induction, consolidation, and maintenance therapy in *FLT3*‐mutated AML (although, as mentioned previously, the FDA did not approve midostaurin for maintenance therapy). One reason was the inability to make definitive conclusions regarding the benefits of maintenance therapy, based on data from older trials using induction and consolidation regimens that are no longer standard; (Other reasons included) missing details from those trials and alternative designs used in those trials. Also noted was the lack of randomized studies demonstrating the efficacy of maintenance therapy after allogeneic HCT.^24^ Nevertheless, maintenance therapy in patients with AML after remission remains an active area of investigation (discussed below).

### 2.3 Post‐HCT relapse prevention

HCT is recommended after induction failure, residual disease, or as post‐remission therapy in properly selected patients.^1^ HCT after first CR can improve the prognosis of patients with AML; however, the possibility of relapse—the leading cause of treatment failure—is significant.^25^, ^26^ Furthermore, relapse after HCT is associated with poor outcomes.^27^ Thus, strategies to avoid relapse following HCT are needed.

Hypomethylating agents, the most commonly used non‐targeted therapies in patients who relapse after HCT, may be effective in preventing post‐HCT relapse by inducing a graft‐vs‐leukemia response through increased expression of tumor antigens.^25^ In a phase 1/2 trial in 27 patients with AML who had undergone a reduced‐intensity conditioning regimen with HCT, azacitidine (36 mg/m^2^ subcutaneously for 5 days in up to 10, 28‐day cycles) after HCT (beginning day 42) induced an increase in circulating regulatory T cells and was well tolerated, without inducing significant graft‐vs‐host disease.^28^ A randomized phase 3 study compared azacitidine (32 mg/m^2^ subcutaneously for 5 days in up to 12, 28‐day cycles; n = 87) vs no further intervention in patients with AML, chronic myelomonocytic leukemia, or MDS after remission with HCT (NCT00887068)^29^; the study was terminated early due to slow accrual. At a median follow‐up of 4.6 years in the azacitidine group and 4.1 years in the control group, no significant effects on relapse‐free survival were observed with azacitidine therapy. However, a nonsignificant trend toward improvement in relapse‐free survival with azacitidine was observed in the subgroup of patients who received at least 9 cycles of treatment. Decitabine (5‐15 mg/m^2^ intravenously for 5 days for up to 8, 6‐week cycles) demonstrated favorable results as post‐HCT maintenance (50‐100 days post‐HCT) in patients with AML (n = 17) or MDS (n = 5). Eight of nine patients who completed study treatment remained in remission through the end of the investigation.^30^ An investigational oral hypomethylating agent, CC‐486, which provides extended dosing to prolong activity, was evaluated in a phase 1/2 study of patients with AML (n = 26) or MDS (n = 4) who had undergone HCT. In 28 evaluable patients, CC‐486 (200‐300 mg orally for 7 days or 150‐200 mg orally for 14 days in up to 12, 28‐day cycles administered post‐HCT) resulted in 1‐year relapse or PFS rates of 54% with a 7‐day regimen, and 72% with a 14‐day regimen. Estimated 1‐year survival rates were 86% and 81%, respectively.^31^

For patients with FLT3‐ITD AML, many FLT3 tyrosine kinase inhibitors are under investigation for post‐HCT maintenance, including sorafenib, midostaurin, quizartinib, crenolanib, and gilteritinib. In an early‐phase study of 22 patients with FLT3‐ITD AML treated with sorafenib following HCT, PFS and OS rates at 1 year were 85% and 95%, respectively.^32^ In a retrospective analysis, patients with FLT3‐ITD AML treated with sorafenib maintenance after HCT during the first CR (n = 26) were compared with matched patients who were not treated with sorafenib (n = 55). The treated patients showed significantly improved 2‐year OS (81% vs 62%, *P* = .029), PFS (82% vs 53%, *P* = .0081), and a lower relapse rate (8.2% vs 37.7%, *P* = .0077) compared with patients not treated with sorafenib.^33^

Midostaurin maintenance was investigated in a phase 2 trial that included 40 patients with newly diagnosed FLT3‐ITD AML post HCT. The trial showed a low incidence of relapse in patients with both high and low FLT3‐ITD mutant to wild type ratio (5% and 12%, respectively).^34^ A randomized, open‐label, phase 2 exploratory trial (RADIUS) compared midostaurin in combination with standard of care vs standard of care in newly diagnosed patients (n = 60) with FLT3‐ITD AML who were in first CR after HCT.^35^ The addition of midostaurin resulted in a 46% relative reduction in the risk of relapse at 18 months. There were estimated relapse rates of 11% and 24% in the midostaurin/standard of care and standard of care groups, respectively; median relapse‐free survival was not reached at 18 months, and follow‐up is ongoing.

A randomized, double blind, placebo‐controlled study (SORMAIN) compared sorafenib maintenance vs placebo in patients (n = 83), with FLT3‐ITD AML in complete hematologic remission after HCT.^36^ At a median follow up of 41.8 months after randomization, median relapse‐free survival was 30.9 months in the placebo group vs not reached in the sorafenib group. This corresponded to a 2‐year relapse‐free survival rate of 53% with placebo vs 85% with sorafenib (*P* = .0135).

Quizartinib was evaluated as maintenance therapy in patients (n = 13) with CR post HCT in a phase 1 study; nine patients survived for at least 50 weeks and 4 patients survived for more than 2 years.^37^ An ongoing phase 3 study (QUANTUM‐R; NCT02039726) includes an evaluation of quizartinib as post‐transplant maintenance, and crenolanib also is under investigation in this setting (NCT02400255). A large, prospective, placebo‐controlled, randomized phase 3 study of nearly 350 patients is underway to determine the benefit of gilteritinib as post‐HCT maintenance therapy in FLT3‐ITD AML during the first CR (NCT02997202). Results of ongoing research will further define the potential benefits of post‐HCT maintenance therapy in patients with AML.

### 2.4 Minimal residual disease and post‐remission therapy

Identification of patients at high risk for disease relapse allows for the use of tailored post‐remission treatment approaches to avoid or delay relapse. Evaluations of minimal residual disease (MRD) after CR with induction therapy are informative because MRD is an important determinant of relapse risk and survival. Methods used to measure MRD include flow cytometry, real‐time quantitative polymerase chain reaction (RT‐qPCR), and targeted next‐generation sequencing (NGS). Use of multiparameter flow cytometry to detect MRD in 186 adults (median age, 51 years; range, 17‐77 years) with AML showed that achievement of MRD− status at CR, during consolidation (median of two cycles, 3‐7 months after initiation), and at completion of intermediate‐dose cytarabine (IDAC) and idarubicin‐based therapy (≥8 months) was highly prognostic for OS. The hazard ratio (95% confidence interval [CI]) for OS was 5.17 (1.98‐13.49) for MRD at CR (*P*  = .0008), 12.57 (3.94‐40.07) for MRD during consolidation (*P* < .0001) 10.19 (2.34‐44.34) for MRD at completion of therapy (*P* = .002).^38^

In a recent study, prospective flow cytometric measurement of MRD after each cycle of standard induction therapy was used to investigate outcomes in a heterogeneous cohort of 2450 patients aged <60 years. After one cycle of induction therapy, patients were divided into risk groups based on a validated system that incorporated cytogenetics, mutation status, and clinical factors. Standard‐risk patients received a second cycle of induction therapy, with a subset subsequently receiving HiDAC consolidation and HCT. The MRD responses in all patients after cycle one predicted OS. The OS in patients with residual disease, partial remission, MRD+, and MRD− were 25%, 36%, 43%, and 63%, respectively (*P* < .0001). Results suggest that this method may help identify patients with standard risk who may benefit from HCT after first remission.^39^ Monitoring of MRD by RT‐qPCR in 346 patients with *NPM1*‐mutated AML (median age, 50 years; range, 6‐68 years) showed MRD in the peripheral blood after two cycles of intensive chemotherapy was highly prognostic for death, with a hazard ratio (95% CI) of 4.84 (2.57‐9.15; *P* < .001). The 5‐year OS rate was 73% in MRD− patients and 24% in MRD+ patients (*P* < .001). Furthermore, no significant benefit of HCT was observed in MRD+ patients in this study, although the number of patients analyzed was small.^40^ In a recent study, targeted NGS carried out during CR to induction therapy in patients with AML frequently detected persistent mutations in *DTA* genes (*DNMT3A*, *TET2*, *ASXL1*), which are associated with age‐related clonal hematopoiesis. These mutations did not correlate with an increased risk of relapse based on 4‐year relapse rates; however, coexisting persistent non‐*DTA* mutations in patients with persistent *DTA* mutations were highly prognostic for relapse (*P* = .002). In addition, the persistence of non‐*DTA* mutations among all patients was associated with an increased risk of relapse (*P* = .001), reduced RFS (*P* = .006), and reduced OS (*P* = .01).^41^ Recently, NGS‐MRD monitoring in AML was shown to be predictive for post‐transplant relapse and survival when used in patients with CR prior to allogeneic HCT,^42^ and to be prognostic for relapse and mortality when used in patients on day 21 after allogenic HCT.^43^

Although MRD negativity is highly prognostic for outcomes in AML,^44^ until recently, a drawback to the use of MRD monitoring was that there was no consensus on determining MRD. Lack of standardization and lack of established cutoff values had limited the widespread use of MRD to guide treatment.^1^ In March 2018, however, flow cytometric, molecular, and clinical MRD recommendations made by consensus of an international panel of experts were published.^44^ Moving forward, these recommendations should provide uniform guidance for the use of MRD to optimize outcomes in AML.

While the prognostic value of MRD is clear, current definitions of CR do not account for MRD, and there are no clear guidelines on how to manage high‐risk patients once they are identified. The impact of MRD on survival was assessed in 359 patients (median age at HCT, 50 years; range, 18‐75 years) in MRD+ CR, MRD− CR, or with active AML before allogeneic HCT. After transplant, the estimated 3‐year OS rate of the 76 MRD+ patients (26%) was similar to that of the 48 patients with active AML (23%), whereas the estimated 3‐year OS rate of the 235 MRD‐ patients was 73%. Multivariable hazard ratio (95% CI) for death was 3.69 (2.51‐5.42) for patients with MRD+ status (*P* < .001) and 4.40 (2.56‐7.55) for patients with active AML (*P* < .001).^45^ These results highlight the prognostic importance of MRD and suggest that morphology‐based assessments of CR alone are not ideal.^45^ In an ongoing phase 2 study (RELAZA2), preemptive treatment with 6 cycles of azacitidine (75 mg/m^2^ × 7 days) and MRD risk‐adapted treatment for up to 18 additional months was evaluated. This was done in patients aged ≥18 years with MRD while in CR after conventional chemotherapy only, or consecutive allogeneic HCT. Preemptive MRD risk‐adapted treatment prevented or substantially delayed disease relapse in 31 of 53 patients who were still in CR after 6 months (58%; 95% CI: 44‐72; *P* < .001).^46^ These results are encouraging; however, future evaluation is needed to identify effective strategies for MRD+ patients.

### 2.5 Future directions: post‐remission therapies under investigation

Selected studies evaluating maintenance therapies in patients with AML are summarized in Table S1. Hypomethylating agents under investigation as maintenance therapies in AML include guadecitabine (SGI‐110) and CC‐486, an oral formulation of azacitidine. Guadecitabine achieved a composite complete response that ranged from 50% to 59% (depending on schedule used) in treatment‐naive patients with AML aged ≥65 years in a randomized, open‐label, phase 1/2 study.^47^ A phase 2 clinical trial involving maintenance therapy (up to 24 months) with guadecitabine with or without idarubicin in previously untreated elderly patients (aged ≥70 years) with AML is ongoing (NCT02096055). CC‐486 is being evaluated as maintenance therapy in a randomized, double‐blind, placebo‐controlled phase 3 trial in patients aged ≥55 years with newly diagnosed AML or AML secondary to prior MDS (NCT01757535). It is hoped that results from this trial will expand treatment options for older patients with AML.^48^

Enasidenib (AG‐221), which is an isocitrate dehydrogenase (IDH) 2 inhibitor approved for use in relapsed or refractory AML with *IDH2* mutation,^49^ is currently being investigated in patients with newly diagnosed AML in a phase 1 trial (NCT02632708). This is in combination with induction therapy (cytarabine + daunorubicin or idarubicin) and consolidation therapy (mitoxantrone + etoposide or cytarabine), and enasidenib may be continued as daily maintenance therapy for a total treatment period of up to 2 years. The trial is also evaluating the IDH1 inhibitor ivosidenib (AG‐120) in a similar treatment plan. Ivosidenib, an oral inhibitor of mutant *IDH1*, is approved by the FDA for the treatment of patients with relapsed/refractory AML with *IDH1* mutation.^50^ A phase 1, multicenter, dose‐escalation study demonstrated an overall response rate of 39.1% and CR rate of 21.8% with ivosidenib 500 mg orally daily in patients with relapsed/refractory AML and *IDH1* mutation (n = 179).^51^ In addition, the NCCN guidelines recommend continuation of ivosidenib therapy post remission until disease progression in patients >60 years of age with *IDH1* mutation who respond to prior lower‐intensity therapy.^1^

FLT3 inhibitors under investigation include quizartinib, crenolanib, and gilteritinib. Quizartinib has shown activity in patients with relapsed/refractory AML and is being investigated in the ongoing QuANTUM‐First randomized, double‐blind, phase 3 study. Evaluation as post‐remission therapy involves the addition of quizartinib or placebo to consolidation therapy (up to 4 cycles of cytarabine) and continued quizartinib or placebo as maintenance therapy for up to 12 cycles in patients with *FLT3*‐mutated AML (NCT02668653). Crenolanib is being studied in combination with consolidation therapy (up to 4 cycles of HiDAC), and continued as maintenance therapy for up to 12 months, in an ongoing trial of patients aged ≤60 years with *FLT3*‐mutated AML.^52^ Gilteritinib (ASP2215) has shown activity in relapsed/refractory *FLT3*‐mutated AML. It is currently being studied in an open‐label, phase 1 trial; evaluation as post‐remission therapy involves administration combination with HiDAC consolidation followed by single‐agent maintenance therapy with gilteritinib once daily, in 28‐day cycles, for up to 26 cycles in patients aged ≥18 years (NCT02236013).^53^

Results of a phase 2 trial demonstrated that sorafenib, a multi‐targeted kinase inhibitor that also inhibits FLT3, added to IDAC consolidation therapy and administered twice daily as single‐agent maintenance therapy in 28‐day cycles for up to 12 cycles in older patients (aged ≥60 years) with *FLT3*‐mutated AML, improved 1‐year OS rates (62% for *FLT3* ITD compared with 30% for elderly historical controls).^54^ In the randomized, phase 2 SORAML trial, sorafenib was added to consolidation therapy in patients aged <60 years with and without mutated *FLT3*. Sorafenib was continued for up to 1 year as maintenance therapy after completion of planned consolidation therapy. Addition of sorafenib resulted in significantly longer event‐free survival (EFS), and a 36% reduction in the risk of relapse or death after prolonged follow‐up, compared with placebo. The 5‐year OS rate showed a trend for improvement with sorafenib; it was 61% with sorafenib vs 50% with placebo.^55^ In contrast, dasatinib did not improve DFS when used as single‐agent maintenance therapy in a phase 2 study in 26 patients (7 with *KIT* mutations; 6 with *FLT*3 mutations; aged 18‐60 years) with core binding factor‐AML in first CR.^56^

Maintenance therapy with immune‐mediated therapies is being investigated in clinical trials. The immune checkpoint inhibitor nivolumab is currently under investigation in ongoing phase 2 clinical trials in patients aged ≥18 years with high‐risk AML, in remission, who were not considered for allogeneic HCT. Preliminary results with nivolumab in patients in CR were reported in 14 patients (median age, 56 years) after a median follow‐up of 11 months (range, 1.4‐26 months) showed 6‐ and 12‐month CR rates of 79% and 71%, respectively, and 12‐ and 18‐month OS rates of 86% and 67%, respectively; the regimen is well tolerated.^57^ Nivolumab is also under investigation in an ongoing randomized phase 2 trial (REMAIN) that is comparing surveillance and single‐agent therapy with nivolumab for AML in patients, post consolidation, who are not candidates for HCT (NCT02275533). Results are expected in June 2019.

The immune stimulator lenalidomide is being evaluated in a phase 2 trial as post‐induction and consolidation therapy in patients with high‐risk AML. Preliminary results with lenalidomide after a median of nine treatment cycles, and a median follow‐up of 19 months (range, 8.5‐39 months), indicate an early signal for improved 6‐month and 12‐month RFS (100% and 69%, respectively) as well as 6‐month and 12‐month OS (100% and 90%, respectively) in 14 patients (median age, 57.5 years; range, 23‐67 years) the regimen is well tolerated.^58^ Lenalidomide is also being evaluated as a maintenance therapy after addition to standard induction therapy in a prospective, randomized, phase 3 study (HOVON 132) in patients aged 18‐65 years; accrual was completed in August 2017 and results are forthcoming.^59^

Combination immunotherapy with histamine dihydrochloride and interleukin‐2 to enhance cytotoxic antileukemic lymphocyte function was investigated in an open‐label, phase 3 trial in 321 adults (median age, 57 years; range, 18‐84 years). Patients in CR were stratified by CR1 or CR >1 and randomized to receive combination therapy or no treatment.^60^ Combination therapy was tolerable and significantly improved leukemia‐free survival vs no treatment ≥3 years after the last patient was enrolled. This was true for all patients (34% vs 24%, respectively; *P* < .01) and for the subset of patients in CR1 at randomization (40% vs 26%, respectively; *P* < .01).

The effects of addition or no addition of the androgen norethandrolone to maintenance therapy with mercaptopurine and methotrexate, during the post‐induction phase, was investigated in a randomized phase 3 study in 325 elderly patients (median age, 70 years) with AML.^61^ All patients received induction therapy with idarubicin, cytarabine, and lomustine. Addition of norethandrolone at 20 mg/day to maintenance therapy for 2 years improved the 5‐year DFS rate (31.2% vs 16.2%; *P* = .002), EFS rate (21.5% vs 12.9%; *P* value not provided), and OS rate (26.3% vs 17.2%; *P* = .008) compared with no addition of norethandrolone.

## 3 CONCLUSIONS

New strategies are needed to prolong remission and improve survival in patients with AML after CR with induction therapy. Traditional consolidation strategies include the use of single‐agent IDAC or HiDAC or cytarabine‐based consolidation regimens, continuing with the same induction regimen that achieved CR, or using allogeneic HCT.^1^ Emerging data suggest that targeted therapy and combinations of chemotherapy and targeted agents may improve outcomes. Research regarding the benefits of maintenance therapy in patients with AML has been inconclusive to date. However, trials to evaluate new agents and new combination therapies as maintenance therapy, including for the prevention of relapse after HCT, are in progress. In addition, given the known heterogeneity of AML, an important goal moving forward is to identify predictive biomarkers of response to specific maintenance therapies. The ultimate goal is to identify regimens that will prolong survival in patients with AML by achieving and maintaining the best response for as long as possible.

## CONFLICT OF INTERESTS

Bruno C. Medeiros ‐ research funds from Astellas, Celgene Corporation, Jazz, and Novartis. Honoraria from Astellas, Celgene Corporation, Jazz, and Novartis.

Steven M. Chan ‐ research funds from Gilead, Celgene Corporation, AbbVie, and Karyopharm. Honorarium from Novartis.

Naval G. Daver: research funds from BMS, Pfizer, Incyte, Servier, AbbVie, Genentech, Immunogen, Nohla Therapeutics, GlycoMimetics, Daiichi‐Sankyo, and Kiromic. Honorarium from Jazz, Pfizer, Otsuka, Celgene Corporation, AbbVie, BMS, Incyte, Immunogen, Agios, Daiichi‐Sankyo, and Novartis.

Brian A. Jonas ‐ research funds to institution from Pharmacyclics, GlycoMimetics, AbbVie, Incyte, Genentech/Roche, Celgene Corporation, Daiichi Sankyo, AROG, Esanex, Forma, Accelerated Medical Diagnostics, and LP Therapeutics. Consultant/Advisory Board for AbbVie, Amgen, Jazz, and Tolero.

Daniel A. Pollyea: research funding from Pfizer, Agios, and AbbVie. Advisory board member for Celgene Corporation, Pfizer, Argenx, Agios, AbbVie, and Celyad.

## Supporting information


**TABLE S1** Summary of clinical trials of maintenance therapies for AML
