# Supplementary material for: Optimizing survival outcomes with post‐remission therapy in acute myeloid leukemia
Source: Am J Hematol. 2019 May 1;94(7):803–11. doi: 10.1002/ajh.25484 (PMC6593671; doi:10.1002/ajh.25484)
Supplement: Supplementary file 1 — TABLE S1 Summary of clinical trials of maintenance therapies for AML [file AJH-94-803-s001.docx]

**Optimizing survival outcomes with post-remission therapy in acute myeloid leukemia**

Bruno C. Medeiros, MD^1^; Steven M. Chan, MD, PhD^2^; Naval G. Daver, MD^3^; Brian A. Jonas, MD, PhD^4^; Daniel A. Pollyea, MD, MS^5^

*^1^Stanford University School of Medicine, Stanford, CA, USA*

*^2^Princess Margaret Cancer Centre, Toronto, ON, Canada*

*^3^Department of Leukemia, MD Anderson Cancer Center, Houston, TX, USA ^4^University of California Davis Comprehensive Cancer Center, Sacramento, CA, USA*

*^5^University of Colorado School of Medicine, Aurora, CO, USA*

**Correspondence:**

Bruno C. Medeiros, MD

Stanford Comprehensive Cancer Center

875 Blake Wilbur Drive

Stanford, CA 94305

brunom@stanford.edu

Phone: (650) 498-6000

Fax: (650) 724-5203

**SUPPLEMENTARY MATERIAL**

**Supplementary Table. Summary of Clinical Trials of Maintenance Therapies for AML**

| **Therapy** | **Study Design** | **End Points** | **Regimens** | **Outcomes** |
| --- | --- | --- | --- | --- |
| Azacitidine^1^ | - Phase 3 - Randomized - Patients aged ≥60 y (n=55) | DFS, OS | Azacitidine maintenance, 50 mg/m^2^, days 1–5, 4-week cycles up to 12 cycles | DFS (vs control):  64% vs 42% (*P* = .04)  OS (vs control):  84% vs 70% (overall)  OS (vs control) after censoring patients with HCT: 82% vs 63% |
| Decitabine^2^ | - Phase 2 (n=134) | DFS, OS | Decitabine maintenance, 20 mg/m^2^, days 1–5, 6-week cycles for 8 cycles | DFS (1- and 3-year)  79%, 54%  OS (1- and 3-year)  96%, 68% |
| Enasidenib^3^ | - Phase 1 - Patients aged ≥18 y - *IDH2* mutation - Relapsed/refractory (n=109) | CR rate, OS | Enasidenib 30, 50, 75, 100, 150 mg BID  Enasidenib 50, 75, 100, 150, 200, 300, 450, 650 mg daily | CR rate, 20.2%  1-year OS, 39% |
| Ivosidenib^4^ | - Phase 1 - Patients aged ≥18 y - *IDH1* mutation - Relapsed/refractory subgroup (n=179) | CR rate, OR rate | Ivosidenib 500 mg once daily | CR rate, 21.8%  OR rate, 39.1% |
| Crenolanib^5^ | - Patients aged ≤60 y - *FLT3* mutation (n=29) | CR rate | Induction  Cytarabine 100 mg/m^2^, days 1–7  Daunorubicin 90 mg/m^2^, days 1–3 or  Idarubicin 12 mg/m^2^, days 1–3  Crenolanib 100 mg TID  Consolidation  HiDAC every 12 h, days 1, 3, 5  Crenolanib 100 mg TID  Maintenance  Crenolanib 100 mg TID up to 12 months | CR rate (overall), 83% |
| Gilteritinib^6^ | - Phase 1 - Open-label - Patients aged ≥18 y - *FLT3* mutation (n=50) | CR rate | Induction  Cytarabine 100 mg/m^2^, days 1–7  Idarubicin 12 mg/m^2^, days 1–3  Gilteritinib 40–120 mg, days 4–17  Consolidation  Cytarabine 1.5 g/m^2^ every 12 h days 1, 3, 5  Gilteritinib 40–120 mg, days 1–14 up to 3 cycles  Maintenance  Gilteritinib once daily, 28-day cycles for up to 26 cycles | CR rate, 57.1% |
| Sorafenib^7^ | - Phase 2 - Randomized - Patients aged ≥60 y - *FLT3* mutation (n=54) | CR rate,  OS | Induction  Cytarabine 100 mg/m^2^, days 1–7  Daunorubicin 60 mg/m^2^, days 1–3  Sorafenib 400 mg BID, days 1–7  Post-remission  Cytarabine 2 g/m^2^, days 1–5  Sorafenib 400 mg BID for 28 days  Maintenance  Sorafenib 400 mg BID for 12 cycles of 28 days | CR rate, 74%  OS 62% (*FLT3* ITD), 71% (*FLT3* TKD), 30% (historical control) |
| Sorafenib^8^ | - Phase 2 - Randomized - Patients aged ≤60 y (n=134 sorafenib/n=133 placebo) | EFS, OS | Induction  Cytarabine 100 mg/m^2^, days 1–7  Daunorubicin 60 mg/m^2^, days 3–5  Sorafenib 400 mg BID or placebo, days 10–19  Consolidation  Cytarabine 3 g/m^2^ BID, days 1, 3, 5  Sorafenib 400 mg BID or placebo, day 8 until 3 days before start of next consolidation  Maintenance  Sorafenib 400 mg BID or placebo | Median RFS (vs placebo), 63 vs 22 months  5-year OS (vs placebo), 61% vs 52% |
| Dasatinib^9^ | - Phase 2 - Open-label - Patients aged 18–60 y - CBF-AML in first CR (n=26) | DFS | Dasatinib maintenance 140 mg once daily for 12 months | 1-year DFS, 31.5%  2-year DFS, 25.7% |
| Nivolumab^10^ | - Phase 2 - Patients aged ≥18 years (n=8) | RFS, OS | Nivolumab maintenance, 3 mg/kg every 2 weeks for 6 months; after 6 months, every 4 weeks until 12 months on study, then every 3 months until relapse | RFS (6- and 12-month)  88%, 73%  OS (6- and 12-month)  100%, 100% |
| Lenalidomide^11^ | - Phase 2 - Patients aged ≥18 y (n=14) | RFS, OS | Lenalidomide maintenance, 10 mg, days 1–28, 28-day cycles up to  24 cycles | RFS (6- and 12-month)  100%, 69%  OS (6- and 12-month)  100%, 90% |
| Histamine dihydrochloride + interleukin-2^12^ | - Phase 3 - Randomized - Open-label - Patients aged ≥18 y (n=160) | LFS | Histamine dihydrochloride 0.5 mg BID for 18 months  Interleukin-2 16,400 U/kg BID for  18 months | 3-year LFS (vs no treatment)  34% vs 24% (*P* < .01) |
| Norethandrolone^13^ | - Phase 3 - Randomized - Open-label - N=325 elderly patients | DFS, EFS  OS, Safety | Induction  Idarubicin 8 mg/m^2^, days 1–5  Cytarabine 100 mg/m^2^, days 1–7  Lomustine 200 mg/m^2^, day 1  Post-induction/maintenance  6 reinduction cycles (1 every 3 months)  Mercaptopurine and methotrexate between reinduction cycles for 2 years  Norethandrolone 10–20 mg/day or no additional treatment for 2 years | 5-year DFS (vs no additional treatment)  31.2% vs 16.2% (*P* = .002)  EFS  21.5% vs 12.9%  OS  26.3% vs 17.2% (*P* = .008) |

BID=twice daily; CBF=core-binding factor; CR=complete remission; DFS=disease-free survival; DOR=duration of response;
EFS=event-free survival; HCT=hematopoietic stem cell transplantation; LFS=leukemia-free survival; MTD=maximum tolerated dose; OR=overall response; OS=overall survival; PK=pharmacokinetics; RFS=relapse-free survival; TID=three times daily; TKD=tyrosine kinase domain.

**References**

1. Huls G, Chitu DA, Havelange V, et al. Azacitidine maintenance after intensive chemotherapy improves DFS in older AML patients. Blood 2019. doi:10.1182/blood-2018-10-879866

2. Blum W, Sanford BL, Klisovic R, et al. Maintenance therapy with decitabine in younger adults with acute myeloid leukemia in first remission: a phase 2 Cancer and Leukemia Group B Study (CALGB 10503). Leukemia 2017;31:34-39.

3. Stein EM, DiNardo CD, Pollyea DA, et al. Enasidenib in mutant-*IDH2* relapsed or refractory acute myeloid leukemia. Blood 2017;130:722-731.

4. DiNardo CD, Stein EM, de Botton S, et al. Durable remissions with ivosidenib in IDH1-mutated relapsed or refractory AML. N Engl J Med 2018;378:2386-2398.

5. Wang E, Tallman M, Stone RM, et al. Low relapse rate in younger patients ≤60 years old with newly diagnosed FLT3-mutated acute myeloid leukemia (AML) treated with crenolanib and cytarabine/anthracycline chemotherapy. Presented at: Blood December 9-12, 2017; Atlanta, GA.

6. Pratz K, Cherry M, Altman JK, et al. Preliminary results from a phase 1 study of gilteritinib in combination with induction and consolidation chemotherapy in subjects with newly diagnosed acute myeloid leukemia (AML) [abstract]. Blood 2017;130(suppl 1):722.

7. Uy GL, Mandrekar SJ, Laumann K, et al. A phase 2 study incorporating sorafenib into the chemotherapy for older adults with FLT3-mutated acute myeloid leukemia: CALGB 11001. Blood Adv 2017;1:331-340.

8. Rollig C, Serve H, Huttmann A, et al. The addition of sorafenib to standard AML treatment results in a substantial reduction in relapse risk and improved survival. Updated results from long-term follow-up of the randomized-controlled soraml trial [abstract]. Blood 2017;130(suppl 1):721.

9. Boissel N, Renneville A, Leguay T, et al. Dasatinib in high-risk core binding factor acute myeloid leukemia in first complete remission: a French Acute Myeloid Leukemia Intergroup trial. Haematologica 2015;100:780-785.

10. Kadia T, Kantarjian H, Jabbour E, et al. Nivolumab maintenance therapy for patients with high-risk acute myeloid leukemia in remission [abstract P211]. Haematologica 2017;102(suppl 2):54.

11. Kadia T, Cortes J, Ravandi F, et al. Lenalidomide maintenance in patients with high risk acute myeloid leukemia [abstract E935]. Haematologica 2017;102(suppl 2):383-384.

12. Brune M, Castaigne S, Catalano J, et al. Improved leukemia-free survival after postconsolidation immunotherapy with histamine dihydrochloride and interleukin-2 in acute myeloid leukemia: results of a randomized phase 3 trial. Blood 2006;108:88-96.

13. Pigneux A, Bene MC, Guardiola P, et al. Addition of androgens improves survival in elderly patients with acute myeloid leukemia: a GOELAMS study. J Clin Oncol 2017;35:387-393.
